# Supplementary material for: Deboronation-Induced Ratiometric Emission Variations of Terphenyl-Based Closo-o-Carboranyl Compounds: Applications to Fluoride-Sensing
Source: Molecules. 2020 May 21;25(10):2413. doi: 10.3390/molecules25102413 (PMC7287808; doi:10.3390/molecules25102413)
Supplement: Supplementary file 1 [file molecules-25-02413-s001.pdf]

Supplementary Material

Deboronation-Induced Ratiometric Emission Variations of Terphenyl-Based *Closo-o*-Carboranyl Compounds: Applications to Fluoride-Sensing

Hyunhee So,[1,†] Min Sik Mun,[1,†] Mingi Kim,[1,†] Jea ho Kim,[1] Ji Hye Lee,[1] Hyonseok Hwang,[1] Duk Keun An,[1,*] and Kang Mun Lee[1,*]

[1] Department of Chemistry, Institute for Molecular Science and Fusion Technology, Kangwon National University, Chuncheon 24341, Republic of Korea; dkan@kangwon.ac.kr (D. K. A.); kangmunlee@kangwon.ac.kr (K.M.L.)

† The first, second and third authors contributed equally to this work.

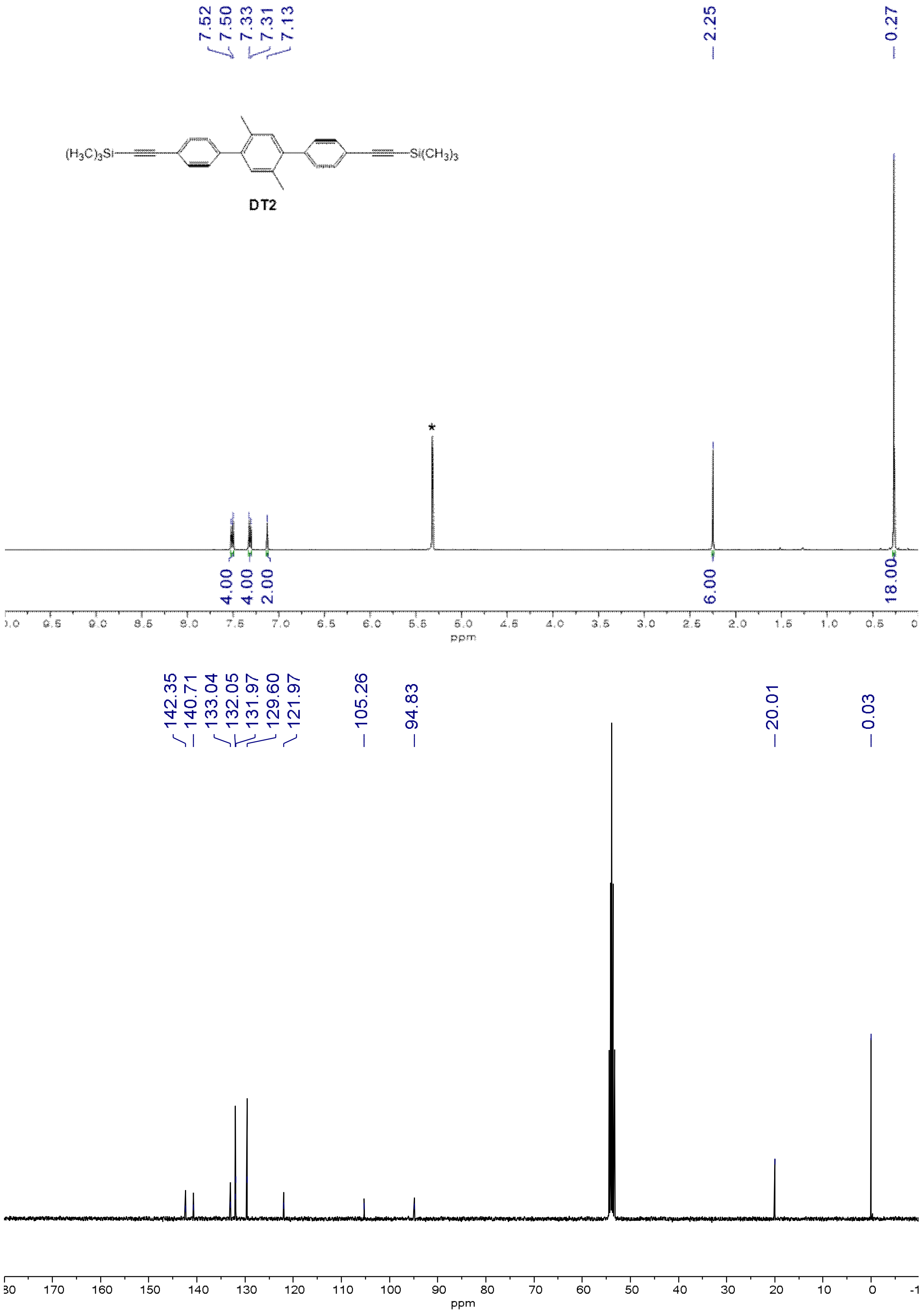

Figure S1. ^{1}H (top) and ^{13}C (bottom) NMR spectra of **DT2** in CD_2Cl_2 (* from residual CH_2Cl_2 in CD_2Cl_2).

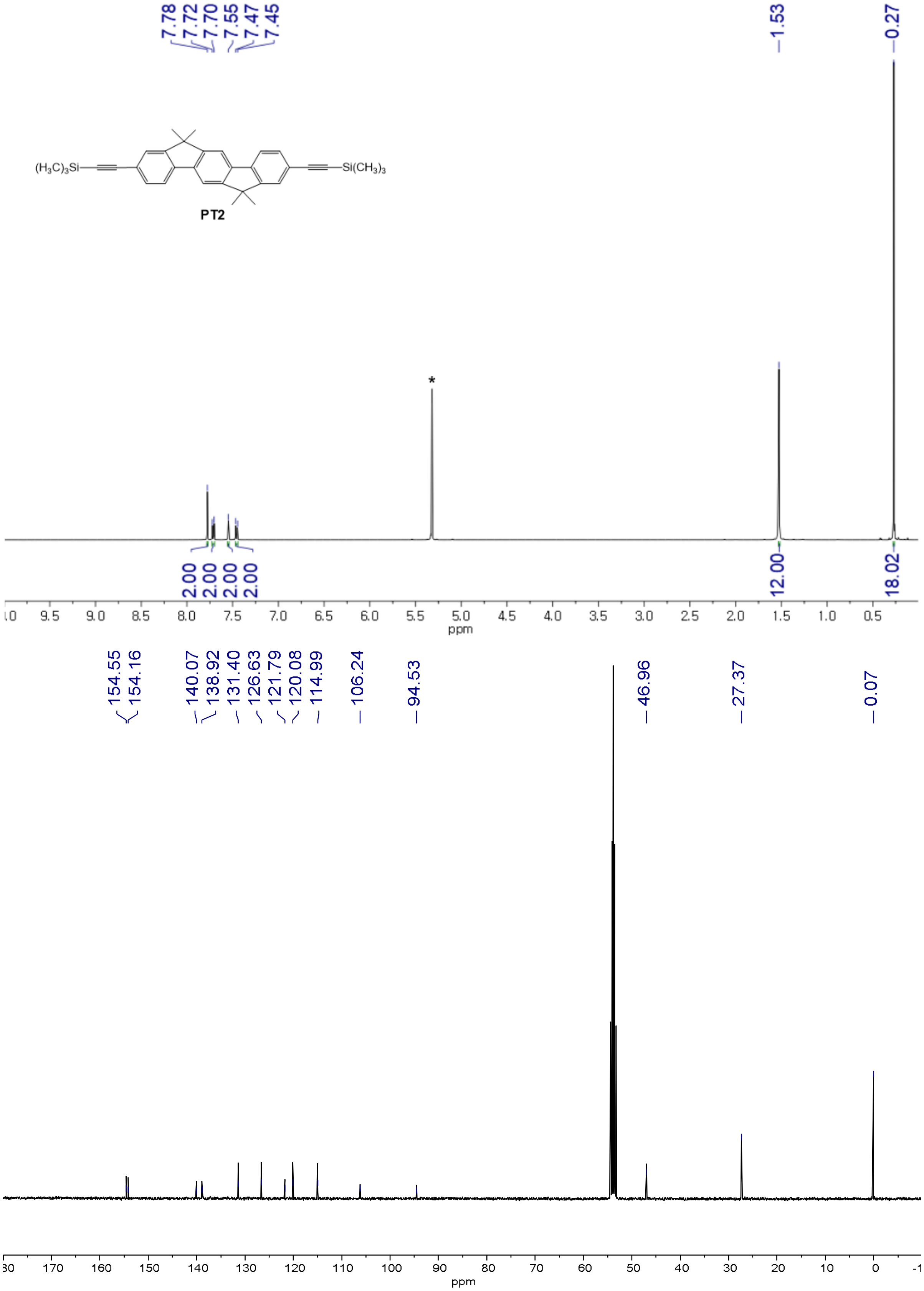

Figure S2. ^{1}H (top) and ^{13}C (bottom) NMR spectra of **PT2** in CD_2Cl_2 (* from residual CH_2Cl_2 in CD_2Cl_2).

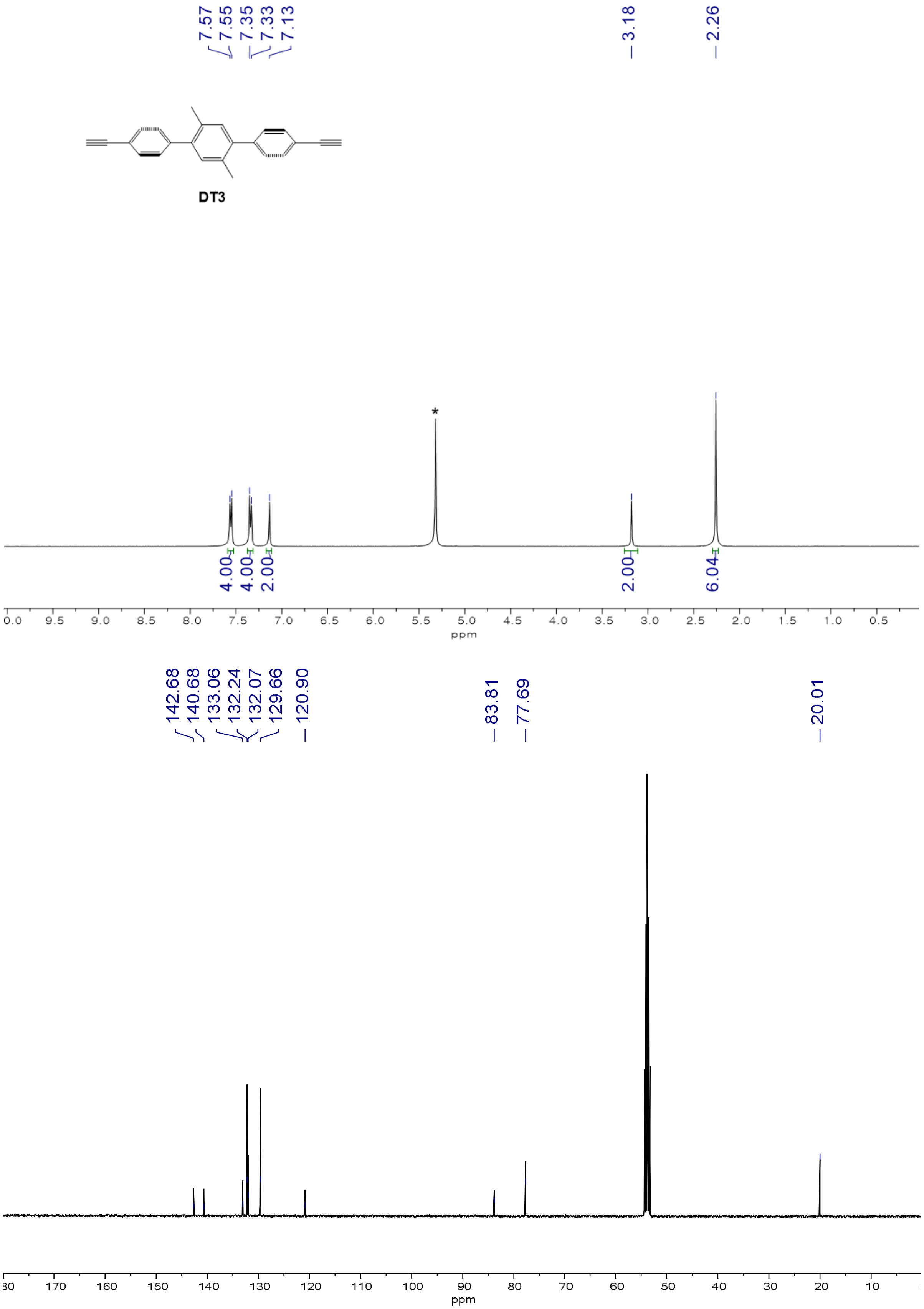

Figure S3. ^{1}H (top) and ^{13}C (bottom) NMR spectra of **DT3** in CD_2Cl_2 (* from residual CH_2Cl_2 in CD_2Cl_2).

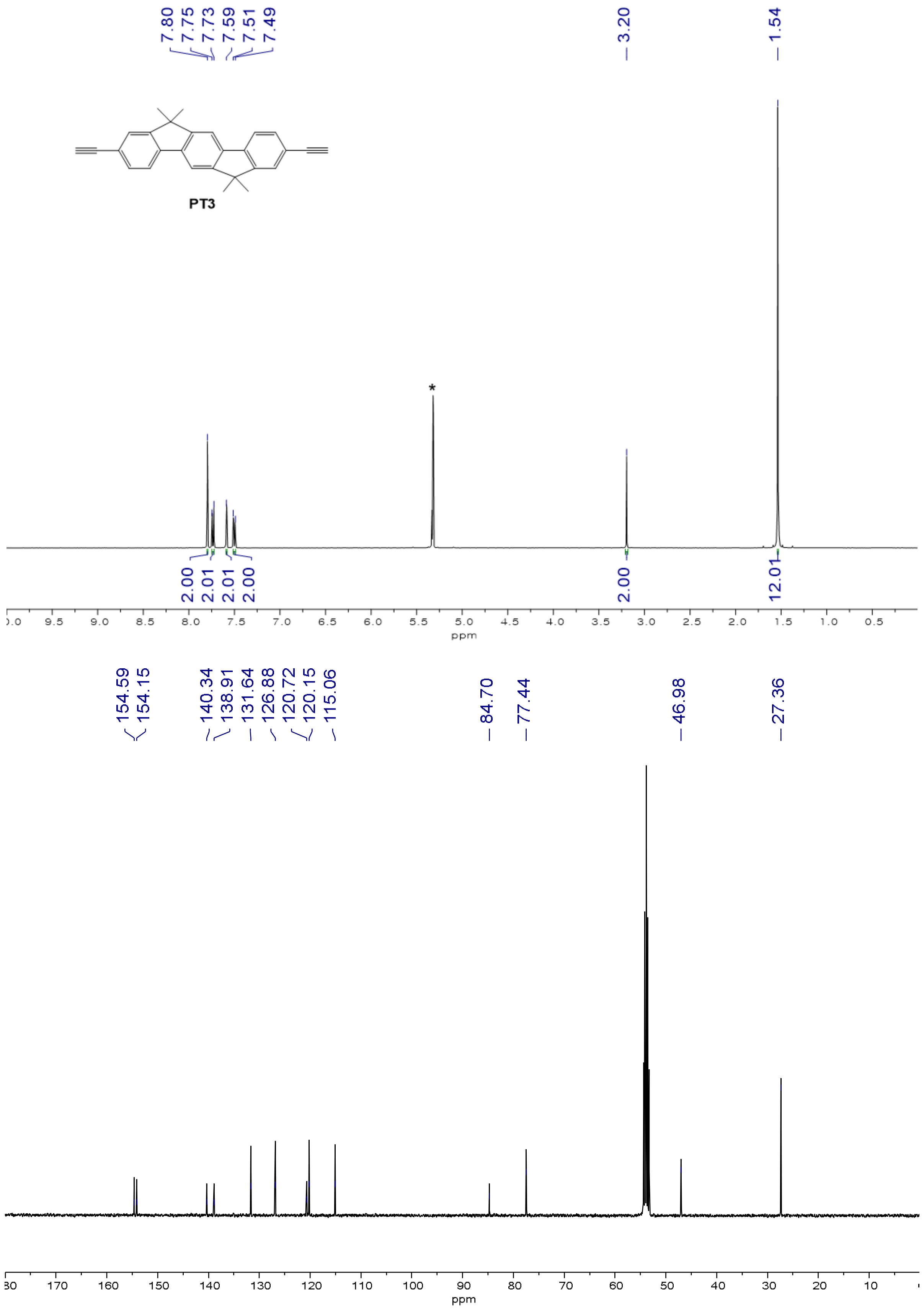

Figure S4. ^{1}H (top) and ^{13}C (bottom) NMR spectra of **PT3** in CD_2Cl_2 (* from residual CH_2Cl_2 in CD_2Cl_2).

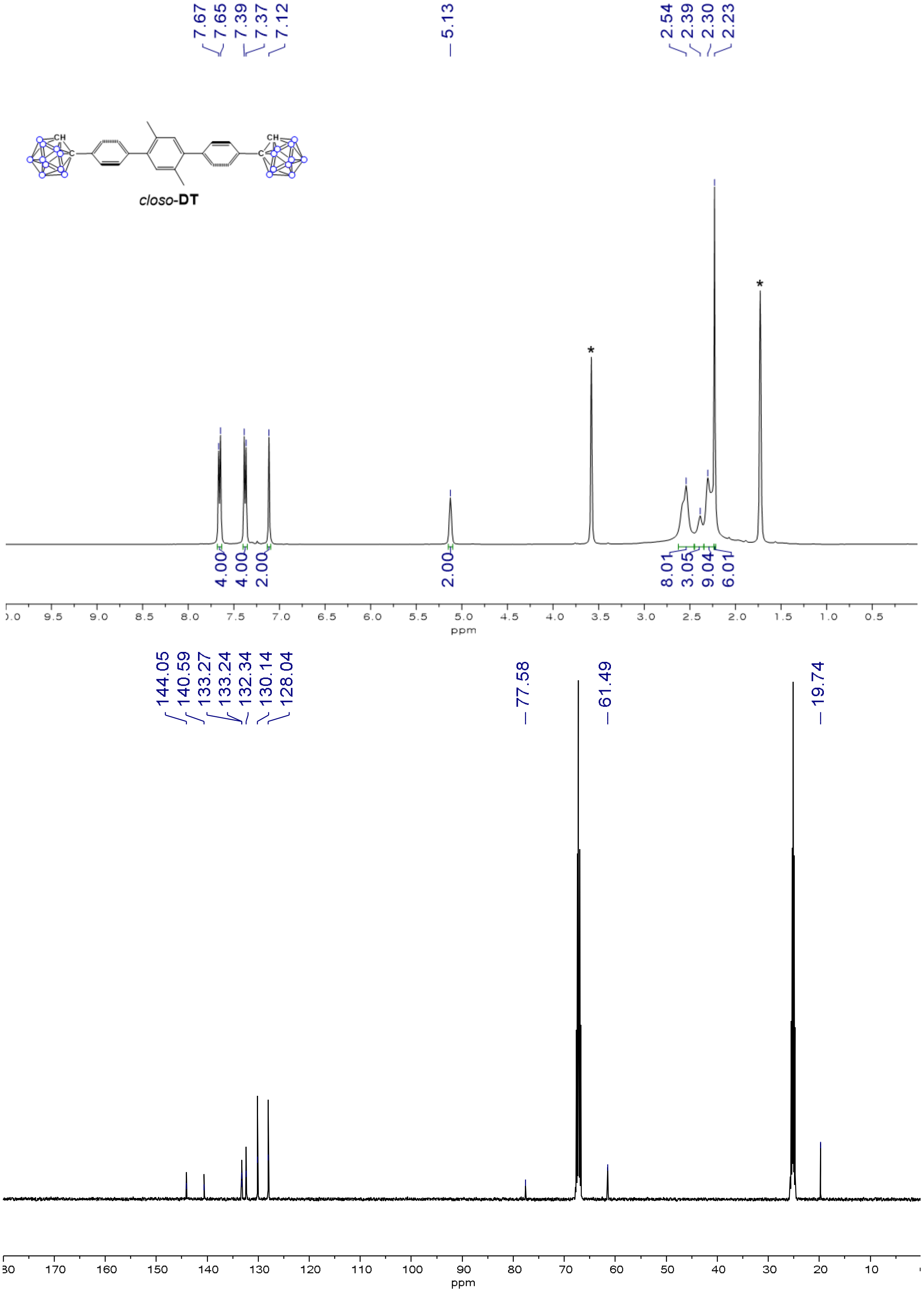

Figure S5. $^{1}H\{^{11}B\}$ (top) and ^{13}C (bottom) NMR spectra of *closo*-**DT** in THF-d_8 (* from residual THF in THF-d_8).

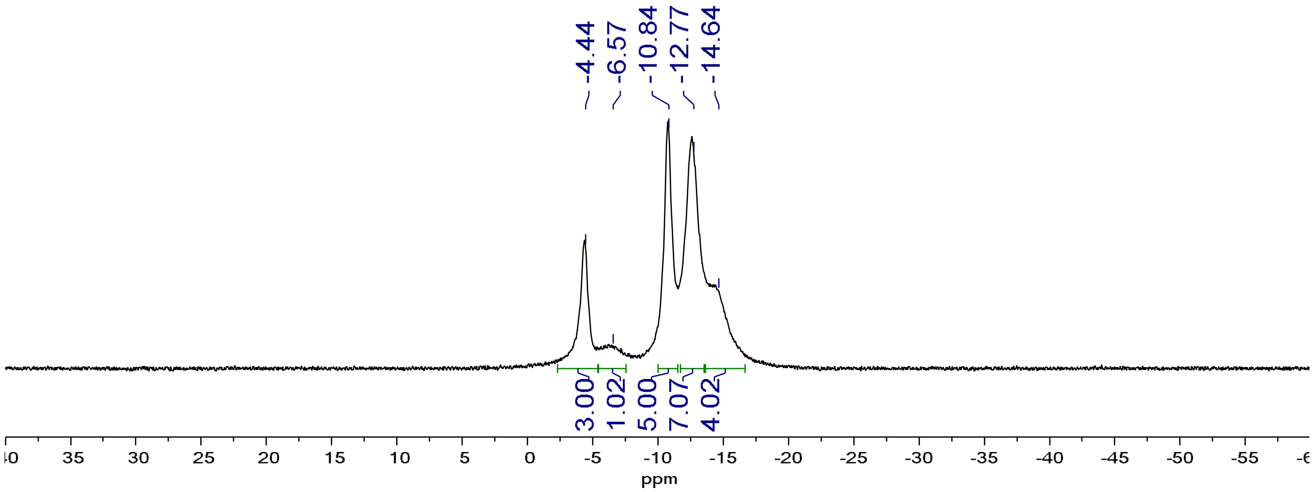

Figure S6. $^{11}B\{^{1}H\}$ NMR spectra of *closo*-**DT** in THF-d_8.

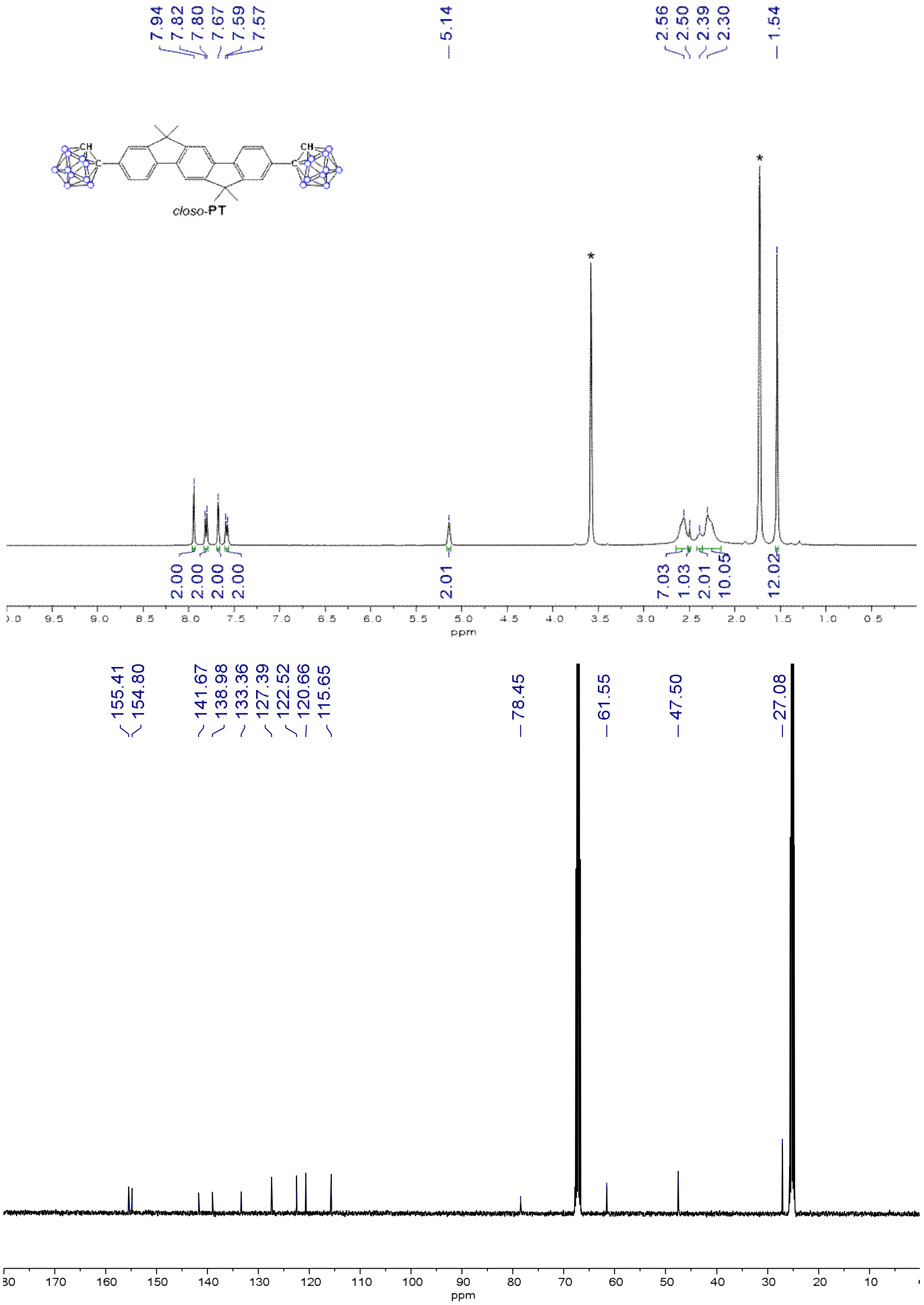

Figure S7. $^{1}H\{^{11}B\}$ (top) and ^{13}C (bottom) NMR spectra of *closo*-**PT** in THF-d_8 (* from residual THF in THF-d_8).

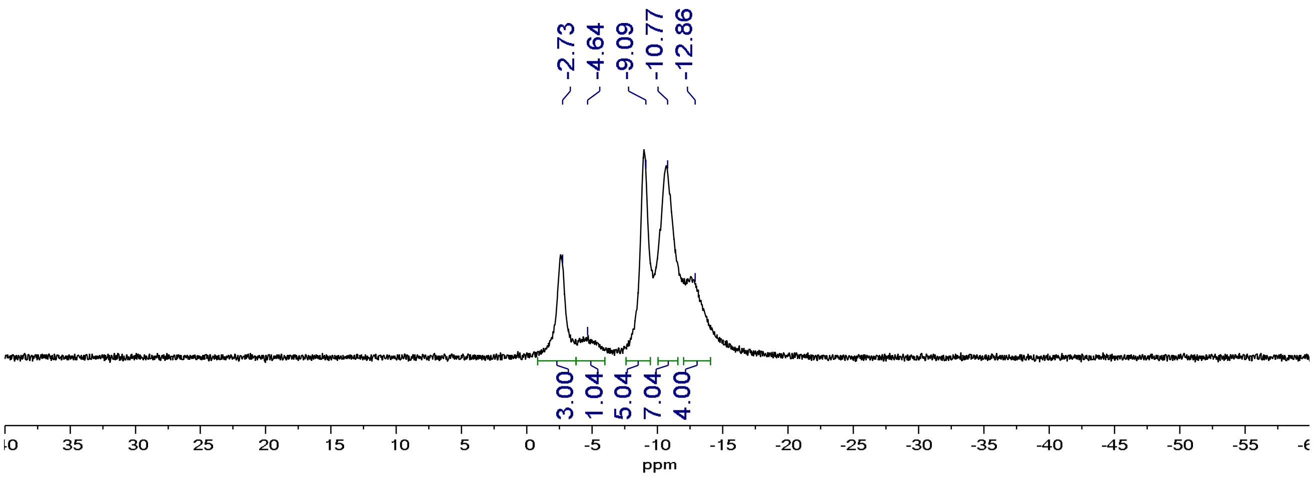

Figure S8. $^{11}B\{^{1}H\}$ NMR spectra of **PT** in THF-d_8.

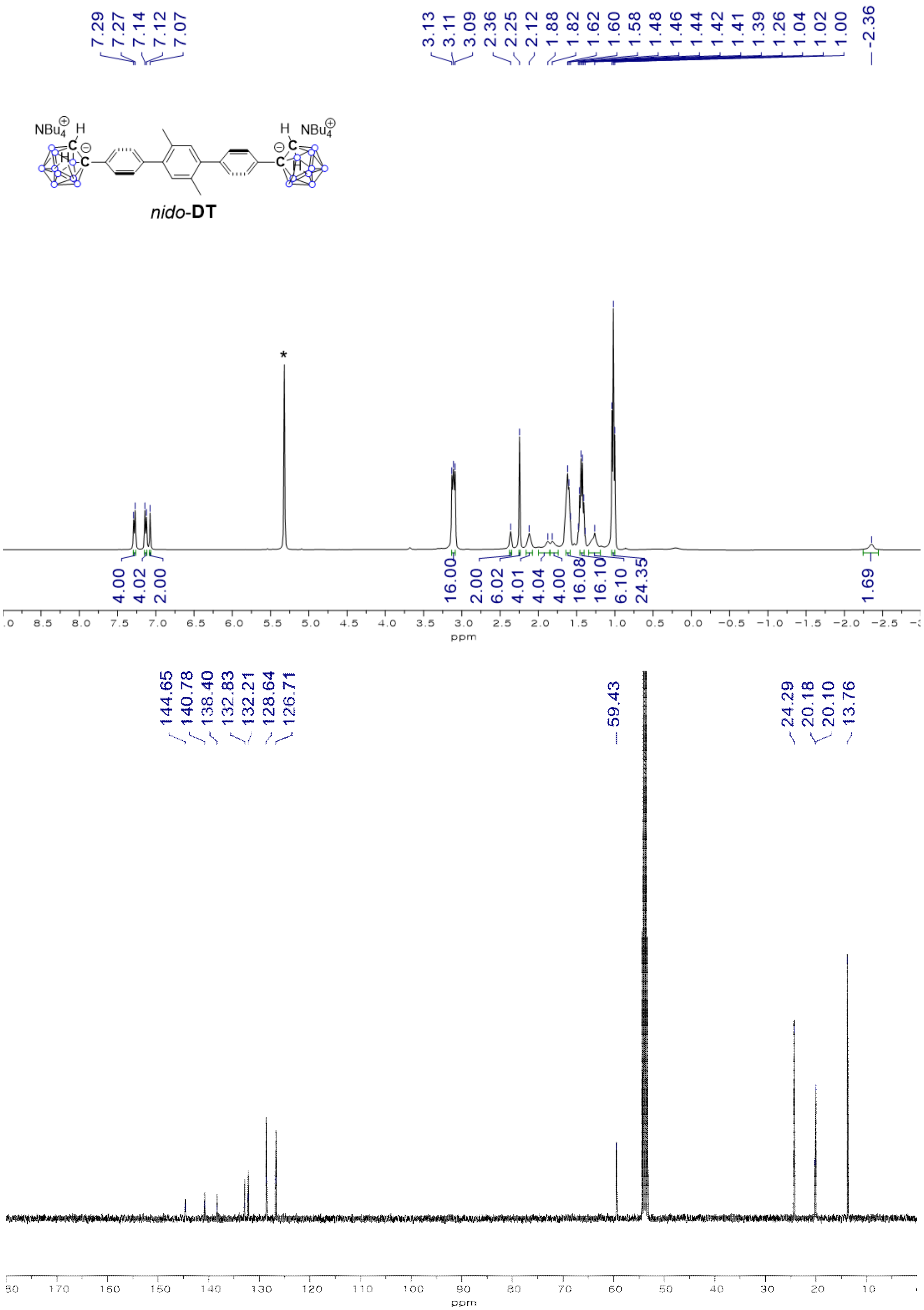

Figure S9. $^{1}H\{^{11}B\}$ (top) and ^{13}C (bottom) NMR spectra of *nido*-**DT** = (*nido*-type of *closo*-**DT**)·$(NBu_4)_2$ in CD_2Cl_2 (* from residual CH_2Cl_2 in CD_2Cl_2).

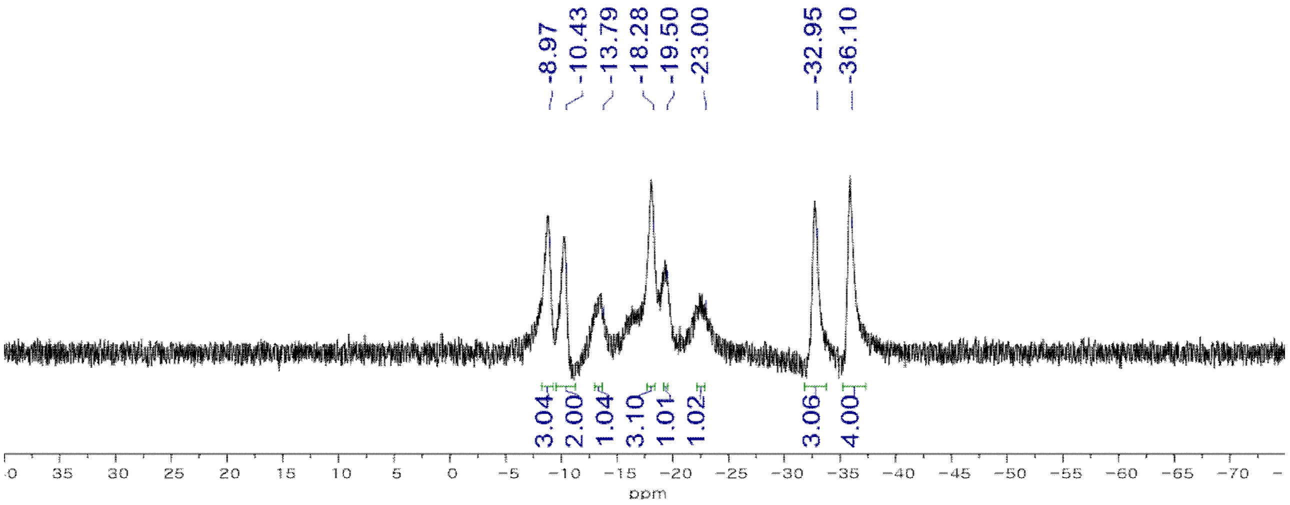

Figure S10. $^{11}B\{^1H\}$ NMR spectra of *nido*-**DT** in CD_2Cl_2.

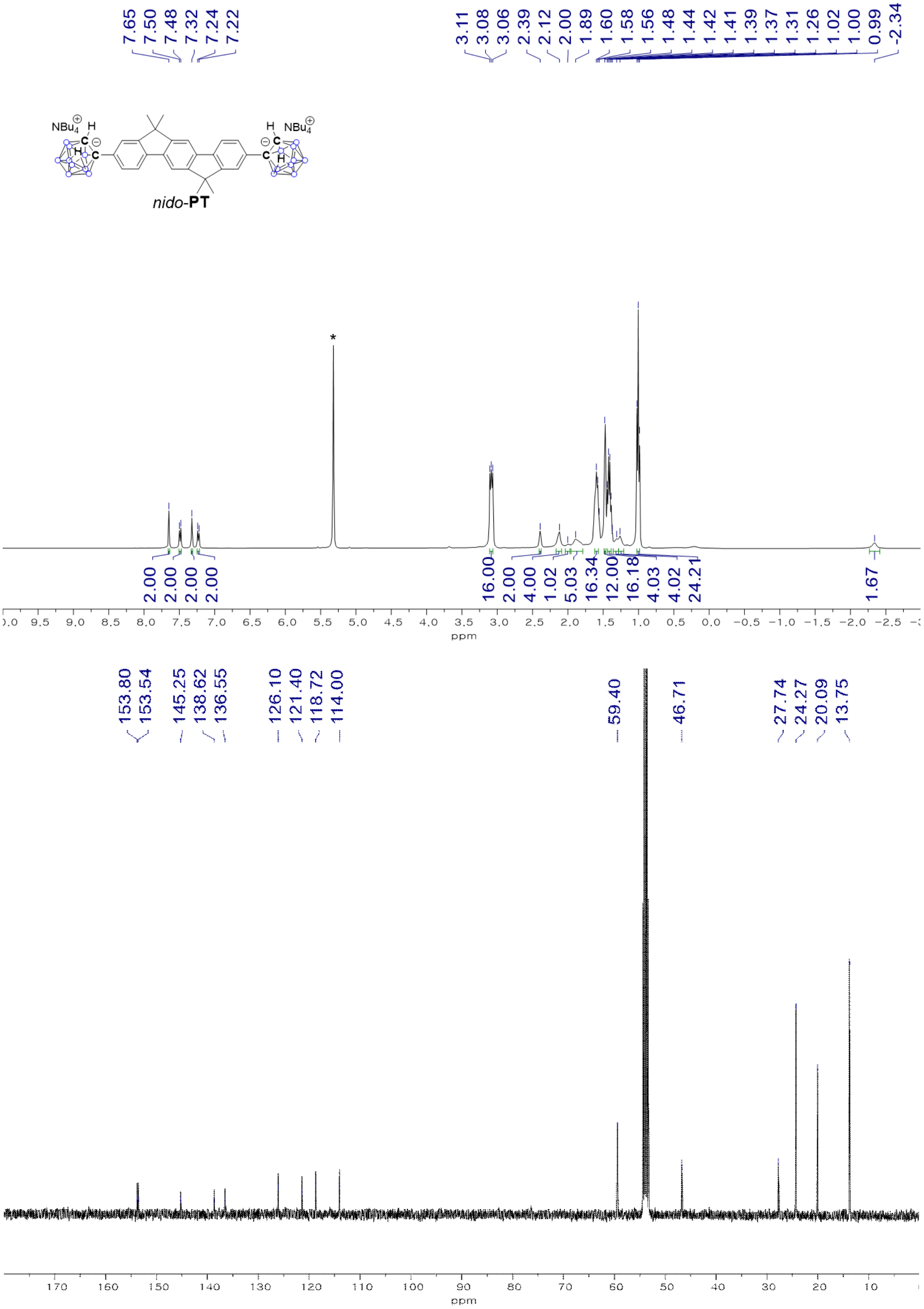

Figure S11. ^{1}H{^{11}B} (top) and ^{13}C (bottom) NMR spectra of *nido*-**PT** = (*nido*-type of *closo*-**PT**)·$(NBu_4)_2$ in CD_2Cl_2 (* from residual CH_2Cl_2 in CD_2Cl_2).

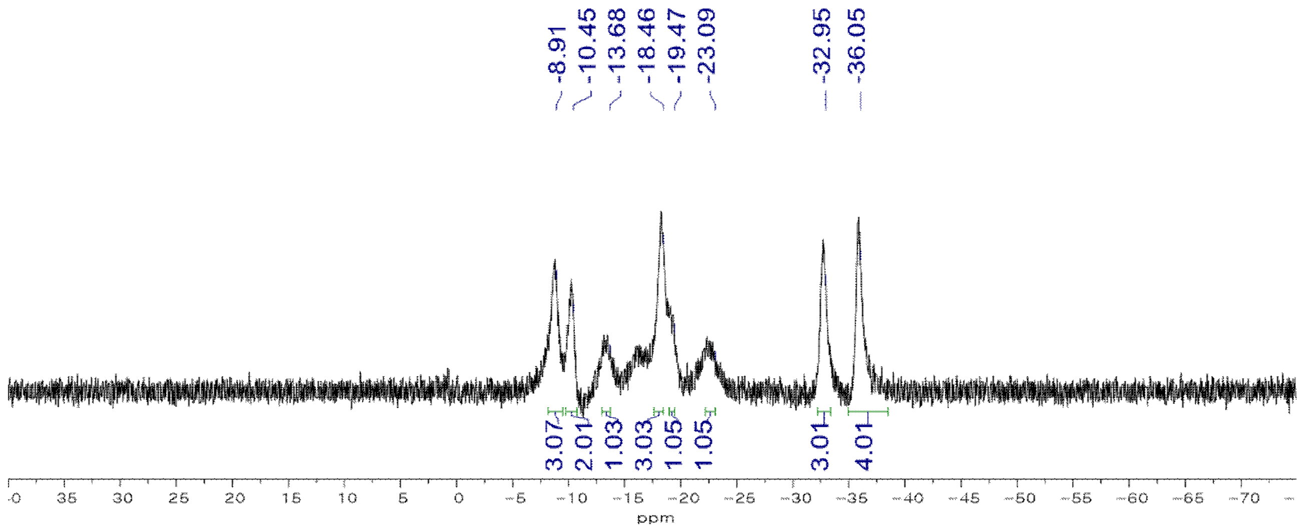

Figure S12. $^{11}B\{^1H\}$ NMR spectra of *nido*-**PT** in CD_2Cl_2.

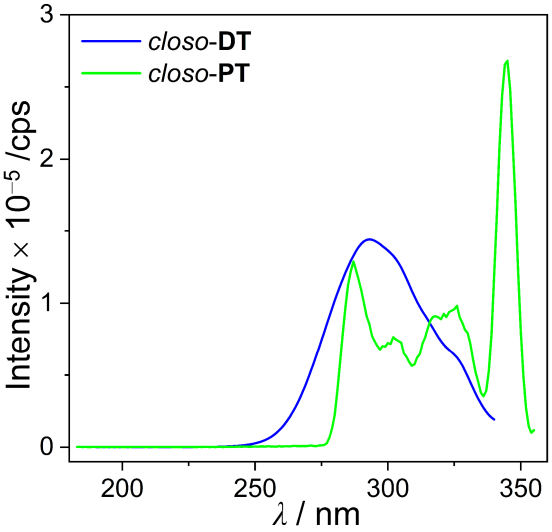

Figure S13. Excitation graphs in THF (30 μM) of *closo*-**DT** and *closo*-**PT**.

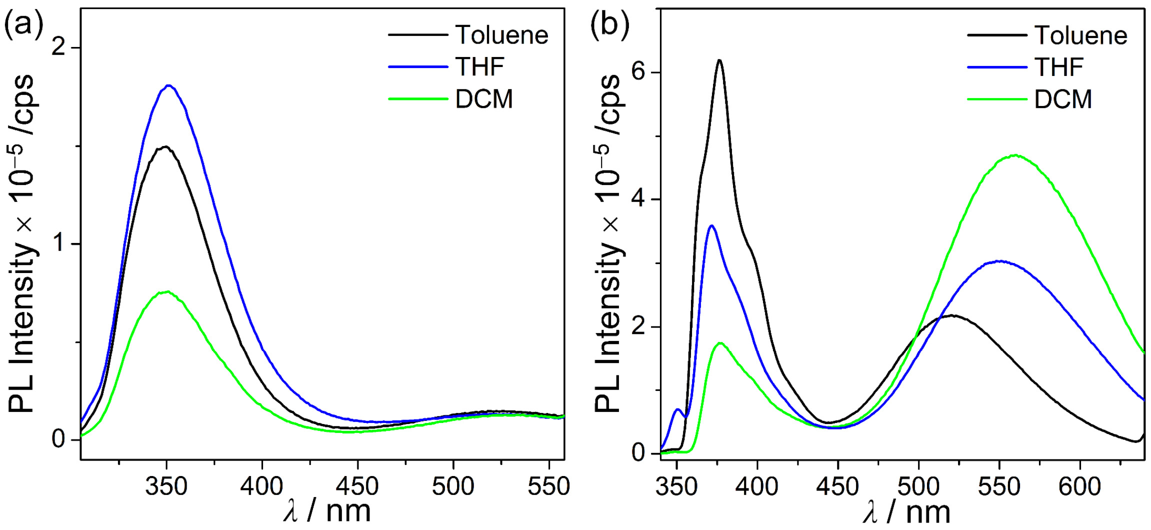

Figure S14. PL spectra of (a) *closo*-**DT** and (b) *closo*-**PT** in various organic solvents (30 μM, λ_{ex} = 292 nm for *closo*-**DT** and 345 nm for *closo*-**PT**).

Computational details

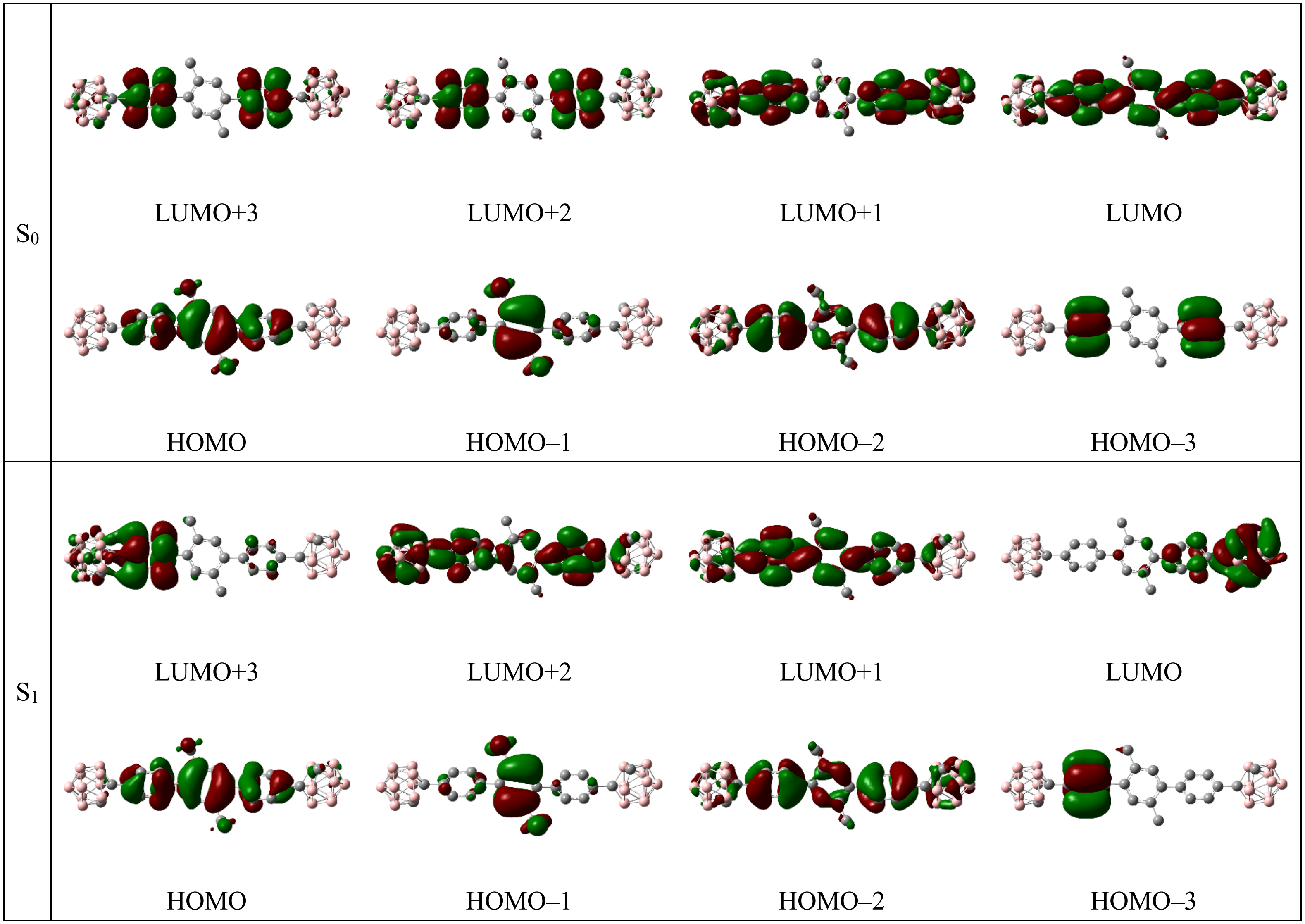

Figure S15. The selected frontier orbitals of *closo*-**DT** from B3LYP calculations (Isovalue = 0.04 a.u.) at the ground state (S_0) and first singlet excited state (S_1) optimized geometries in THF.

Table S1. Computed absorption wavelengths (λ_{calc} in nm) and oscillator strengths ($f_{calc.}$) for *closo*-**DT** from TD-B3LYP calculations using the B3LYP geometries at the ground state (S_0) and first singlet excited state (S_1) optimized geometries in THF

state	λ_{calc} (/nm)	f_{calc}	Major contribution
			S_0
1	285.70	1.2315	HOMO → LUMO (98.0%)
2	274.14	0.1986	HOMO−1 → LUMO (85.6%)
			HOMO → LUMO+5 (8.5%)
3	264.19	0.0000	HOMO → LUMO+1 (96.3%)
4	255.75	0.0000	HOMO−3 → LUMO (23.1%)
			HOMO → LUMO+3 (64.0%)
5	255.56	0.0052	HOMO−4 → LUMO (23.2%)
			HOMO → LUMO+2 (61.4%)
			S_1
1	509.37	0.5920	HOMO → LUMO (99.6%)
2	426.04	0.0568	HOMO−1 → LUMO (99.1%)
3	383.52	0.0196	HOMO−3 → LUMO (88.5%)
			HOMO−2 → LUMO (8.0%)
4	359.14	0.2721	HOMO−4 → LUMO (8.1%)
			HOMO → LUMO+1 (87.7%)
5	343.53	0.0004	HOMO−5 → LUMO (96.2%)

Table S2. Molecular orbital energies (in eV) and molecular orbital distributions (in %) of *closo*-**DT** at the ground state (S_0) and first singlet excited state (S_1) optimized geometries in THF

	E (eV)	carborane	terphenyl
		S_0	
LUMO+3	−0.64	12.8	87.2
LUMO+2	−0.65	12.1	87.9
LUMO+1	−1.02	37.7	62.3
LUMO	−1.48	17.0	83.0
HOMO	−6.22	3.0	97.0
HOMO−1	−6.66	0.7	99.3
HOMO−2	−7.11	10.4	89.6
HOMO−3	−7.28	0.9	99.1
		S_1	
LUMO+3	−0.63	11.4	88.6
LUMO+2	−0.75	31.9	68.1
LUMO+1	−1.62	13.7	86.3
LUMO	−3.48	82.8	17.2
HOMO	−6.03	5.7	94.3
HOMO−1	−6.78	0.8	99.2
HOMO−2	−7.12	13.7	86.3
HOMO−3	−7.33	0.9	99.1

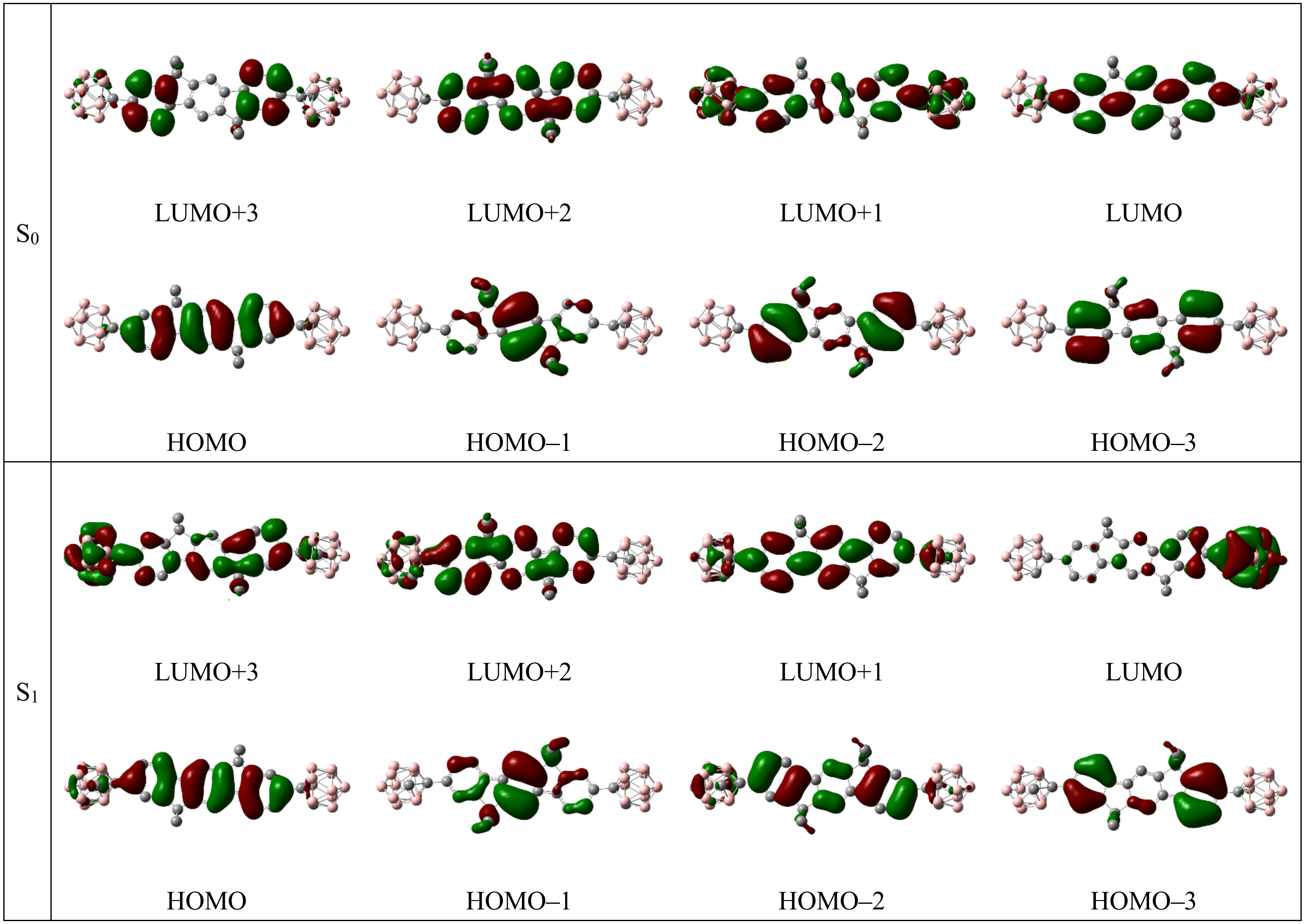

Figure S16. The selected frontier orbitals of *closo*-**PT** from B3LYP calculations (Isovalue = 0.04 a.u.) at the ground state (S_0) and first singlet excited state (S_1) optimized geometries in THF.

Table S3. Computed absorption wavelengths (λ_{calc} in nm) and oscillator strengths ($f_{calc.}$) for *closo*-**PT** from TD-B3LYP calculations using the B3LYP geometries at the ground state (S_0) and first singlet excited state (S_1) optimized geometries in THF

state	λ_{calc} (/nm)	f_{calc}	Major contribution
			S_0
1	348.17	1.7222	HOMO → LUMO (98.8%)
2	289.43	0.0320	HOMO−3 → LUMO (7.3%)
			HOMO−1 → LUMO (41.4%)
			HOMO → LUMO+2 (43.9%)
3	278.75	0.0000	HOMO−2 → LUMO (25.6%)
			HOMO → LUMO+1 (54.3%)
			HOMO → LUMO+3 (16.0%)
4	278.47	0.0386	HOMO−3 → LUMO (16.8%)
			HOMO−1 → LUMO (41.3%)
			HOMO → LUMO+2 (29.4%)
5	271.81	0.0000	HOMO−4 → LUMO (12.5%)
			HOMO−2 → LUMO (14.2%)
			HOMO → LUMO+1 (38.9%)
			HOMO → LUMO+3 (29.0%)
			S_1
1	554.44	0.9435	HOMO → LUMO (99.7%)
2	395.36	0.0163	HOMO−4 → LUMO (16.1%)
			HOMO−3 → LUMO (7.1%)
			HOMO−2 → LUMO (14.0%)
			HOMO−1 → LUMO (61.7%)
3	381.97	0.0031	HOMO−4 → LUMO (28.4%)
			HOMO−2 → LUMO (28.9%)
			HOMO−1 → LUMO (36.7%)
4	371.34	0.4002	HOMO−4 → LUMO (7.2%)
			HOMO−3 → LUMO (7.4%)
			HOMO → LUMO+1 (78.9%)
5	352.54	0.0002	HOMO−5 → LUMO (97.7%)

Table S4. Molecular orbital energies (in eV) and molecular orbital distributions (in %) of *closo*-**PT** at the ground state (S_0) and first singlet excited state (S_1) optimized geometries in THF.

	E (eV)	carborane	terphenyl
		S_0	
LUMO+3	−0.53	12.4	87.6
LUMO+2	−0.75	7.1	92.9
LUMO+1	−0.88	33.8	66.2
LUMO	−1.83	16.1	83.9
HOMO	−5.83	3.8	96.2
HOMO−1	−6.84	0.1	99.9
HOMO−2	−7.06	2.3	97.7
HOMO−3	−7.13	1.0	99.0
		S_1	
LUMO+3	−0.64	28.9	71.1
LUMO+2	−0.80	11.6	88.4
LUMO+1	−1.77	14.0	86.0
LUMO	−3.17	80.2	19.8
HOMO	−5.74	8.0	92.0
HOMO−1	−6.96	0.2	99.8
HOMO−2	−7.07	9.8	90.2
HOMO−3	−7.22	4.2	95.8

Table S5. Cartesian coordinates of the ground state (S_0) fully optimized geometry in THF of *closo*-**DT** from B3LYP calculations (in Å)

Atom	x	y	z
C	-8.023338	-1.268662	-0.509786
H	-10.145290	1.488057	-2.063366
H	-7.357125	0.143366	-2.466986
H	-9.767760	-1.596815	-2.247668
H	-11.692112	-0.326012	-0.128903
H	-7.593454	2.468360	-0.583949
H	-9.773424	-2.720121	0.491396
H	-10.311893	2.213974	0.916024
H	-7.345356	-1.637402	1.863402
H	-7.634531	1.345816	2.171509
H	-10.143679	-0.385283	2.525425
B	-8.042273	0.128041	-1.508281
B	-9.619717	0.853169	-1.210486
B	-9.466780	-0.915150	-1.329159
B	-8.182584	1.468753	-0.355674
B	-10.512638	-0.206352	-0.078996
B	-9.472364	-1.589458	0.318491
B	-9.712859	1.267379	0.524032
B	-8.040488	-0.977464	1.176616
B	-8.185377	0.787477	1.288989
B	-9.618835	-0.247549	1.470837
C	-5.738968	0.084775	0.030961
C	-3.639949	0.863084	0.971234
C	-5.008915	-0.646180	-0.916320
H	-3.124035	1.443204	1.728545
H	-5.513877	-1.225195	-1.681557
C	-7.249732	0.078882	0.036808
C	-5.032202	0.841077	0.975132
H	-2.289377	1.413955	1.723776
H	-7.381091	-2.077511	-0.831315
C	-2.900741	0.129407	0.030659
C	-3.616901	-0.627627	-0.910190
H	-3.077616	-1.195093	-1.661881
C	-1.411008	0.109248	0.036261
C	-0.764267	-1.135730	0.022171
C	-0.625567	1.283162	0.029496
C	0.625545	-1.283056	0.029498
H	-1.376884	-2.033520	0.014168
C	0.764248	1.135837	0.022279
C	1.410989	-0.109140	0.036370
H	1.376873	2.033623	0.014354
C	2.900732	-0.129299	0.030840
C	3.616892	0.627750	-0.909999
C	3.639948	-0.862963	0.971416
C	5.008907	0.646293	-0.916155
H	3.077595	1.195246	-1.661657
C	5.032201	-0.840968	0.975282
H	3.124054	-1.443062	1.728756
C	5.738971	-0.084706	0.031080
H	5.513849	1.225354	-1.681368
H	5.564938	-1.413826	1.723941
C	7.249742	-0.078874	0.036868
B	8.042159	-0.128004	-1.508291
B	8.182458	-1.468791	-0.355762
B	8.185423	-0.787611	1.288946
H	7.356907	-0.143201	-2.466919
B	9.619575	-0.853265	-1.210643
B	9.466764	0.915078	-1.329206
H	7.593218	-2.468329	-0.584053
B	9.712810	-1.267586	0.523844
H	7.634589	-1.345927	2.171486
B	8.040646	0.977366	1.176674
B	9.618964	0.247298	1.470745
H	10.145033	-1.488116	-2.063621
B	10.512654	0.206124	-0.079173
C	8.023403	1.268657	-0.509698
H	9.767712	1.596759	-2.247713
B	9.472515	1.589288	0.318493
H	10.311796	-2.214261	0.915716
H	7.345603	1.637288	1.863564
H	10.143889	0.384946	2.525303
H	11.692134	0.325723	-0.129109
H	7.381208	2.077579	-0.831144
H	9.773653	2.719928	0.491412
C	-1.214285	2.676407	-0.001744
H	-2.131056	2.719768	-0.595260
H	-1.463232	3.038239	1.002760
H	-0.497462	3.383080	-0.428516
C	1.214262	-2.676299	-0.001726
H	2.130962	-2.719692	-0.595355
H	1.463342	-3.038065	1.002766
H	0.497390	-3.383000	-0.428367

Table S6. Cartesian coordinates of the first excited state (S_1) fully optimized geometry in THF of *closo*-**DT** from B3LYP calculations (in Å)

Atom	x	y	z
C	-8.088288	-1.294836	-0.299219
H	-10.186412	1.364047	-2.043533
H	-7.441031	-0.100619	-2.399569
H	-9.898239	-1.737899	-1.939981
H	-11.714220	-0.213940	0.105475
H	-7.560216	2.401674	-0.744351
H	-9.834958	-2.592853	0.892132
H	-10.235856	2.374070	0.855388
H	-7.334348	-1.454498	2.076990
H	-7.533318	1.546388	2.105846
H	-10.079403	-0.064660	2.703804
B	-8.104879	-0.002173	-1.430478
B	-9.650176	0.796748	-1.151408
B	-9.550015	-0.979166	-1.102866
B	-8.171710	1.449133	-0.402956
B	-10.531693	-0.123682	0.106284
B	-9.513504	-1.492728	0.602111
B	-9.675697	1.377295	0.539656
B	-8.038861	-0.843744	1.355436
B	-8.137785	0.929319	1.300982
B	-9.587996	-0.042525	1.625284
C	-5.761278	0.062461	0.033435
C	-3.643341	1.054691	0.683608
C	-5.059224	-0.875448	-0.742219
H	-3.113547	1.790709	1.273696
H	-5.584464	-1.610676	-1.339590
C	-7.267889	0.075317	0.091877
C	-5.028100	1.025231	0.746504
H	-5.541162	1.755267	1.358758
H	-7.486413	-2.154336	-0.559732
C	-2.925375	0.104666	-0.076874
C	-3.673921	-0.868231	-0.780384
H	-3.163762	-1.592625	-1.404733
C	-1.462150	0.078516	-0.121016
C	-0.817884	-1.180518	-0.109028
C	-0.645938	1.266535	-0.201939
C	0.555859	-1.341572	-0.096734
H	-1.435726	-2.070917	-0.069819
C	0.728201	1.102244	-0.220603
C	1.374743	-0.152193	-0.135437
H	1.348684	1.989842	-0.276747
C	2.843000	-0.180727	-0.127795
C	3.569646	0.689714	-0.972471
C	3.580554	-1.022654	0.733511
C	4.956752	0.699510	-0.971053
H	3.037772	1.332734	-1.666247
C	4.967404	-0.988327	0.753624
H	3.063998	-1.673398	1.428686
C	5.691234	-0.130083	-0.099503
H	5.484323	1.359302	-1.650232
H	5.503969	-1.631733	1.441219
C	7.172190	-0.107594	-0.088103
B	8.066261	1.357246	-0.350107
B	8.098348	-0.156715	-1.423574
B	8.109086	-1.434797	-0.144950
H	7.467351	2.315475	-0.718417
B	9.581040	0.782094	-1.244878
B	9.794184	1.589030	0.315997
H	7.532647	-0.215001	-2.467763
B	9.613211	-0.997200	-0.999173
H	7.551713	-2.483960	-0.195952
B	8.083269	-0.364056	1.370434
B	9.599703	-1.243986	0.780040
H	10.038872	1.294421	-2.217645
B	10.611888	0.030321	0.019198
C	8.533206	1.268441	1.279648
H	10.303125	2.661047	0.388474
B	9.807468	0.317911	1.586158
H	10.105529	-1.762146	-1.769062
H	7.496991	-0.739044	2.333741
H	10.070999	-2.213242	1.286777
H	11.800571	-0.022599	-0.045373
H	8.125605	1.975057	1.990516
H	10.326494	0.394495	2.653052
C	-1.209307	2.658521	-0.324268
H	-2.128828	2.681722	-0.912196
H	-1.434762	3.088599	0.658721
H	-0.477932	3.315917	-0.798852
C	1.126846	-2.734098	-0.093894
H	2.018007	-2.808459	-0.720613
H	1.413263	-3.050195	0.915916
H	0.382330	-3.446981	-0.454738

Table S7. Cartesian coordinates of the ground state (S_0) fully optimized geometry in THF of *closo*-**PT** from B3LYP calculations (in Å)

Atom	x	y	z
C	7.943384	0.485214	-1.347734
H	9.626059	-2.625519	0.064097
H	6.993910	-1.813610	-1.395069
H	9.578270	-0.947790	-2.551135
H	11.508341	-0.207458	-0.198511
H	7.128498	-1.688888	1.625996
H	9.905578	1.923694	-1.840920
H	9.952129	-0.674333	2.408231
H	7.542908	2.704848	-0.281369
H	7.448110	1.176802	2.329460
H	10.179163	2.158648	1.240780
B	7.734441	-1.116228	-0.800899
B	9.240942	-1.504129	0.029431
B	9.278912	-0.557445	-1.475408
B	7.822070	-0.991641	0.970145
B	10.329797	-0.091971	-0.121348
B	9.473267	1.155204	-1.052542
B	9.428502	-0.360252	1.390334
B	8.054167	1.658770	-0.112137
B	8.018074	0.725452	1.396855
B	9.563685	1.288681	0.719954
C	5.558760	0.384368	0.031730
C	3.552823	1.771968	0.051021
C	4.753377	-0.771038	0.036731
C	2.764191	0.620481	0.045412
H	3.103806	2.760335	0.062481
H	5.212366	-1.752814	0.041532
C	7.064084	0.242337	0.054385
C	2.428093	-2.645504	-1.213702
H	3.383672	-3.179409	-1.230057
H	1.627065	-3.391361	-1.227232
H	2.353597	-2.045872	-2.125378
C	2.433101	-2.635008	1.317445
H	1.632441	-3.381046	1.339804
H	3.389227	-3.167825	1.335152
H	2.361535	-2.027623	2.224074
C	4.941562	1.646731	0.048255
H	5.543968	2.545407	0.065126
C	3.372033	-0.650441	0.042761
C	1.030072	-0.940065	0.048135
C	2.320959	-1.759615	0.048316
C	1.309505	0.445491	0.047706
C	0.282408	1.397837	0.048321
H	0.508240	2.460837	0.048762
C	-0.282408	-1.397837	0.048320
H	-0.508240	-2.460837	0.048759
C	-1.030072	0.940065	0.048136
C	-1.309505	-0.445491	0.047705
C	-2.764191	-0.620481	0.045412
C	-3.552823	-1.771968	0.051021
C	-3.372033	0.650441	0.042762
C	-4.941562	-1.646731	0.048255
H	-3.103806	-2.760335	0.062479
C	-4.753377	0.771038	0.036732
C	-5.558760	-0.384368	0.031731
H	-5.543968	-2.545407	0.065125
H	-5.212366	1.752814	0.041535
C	-2.320959	1.759615	0.048318
C	-2.433100	2.635007	1.317449
H	-2.361534	2.027621	2.224077
H	-1.632440	3.381044	1.339808
H	-3.389227	3.167824	1.335156
C	-2.428093	2.645506	-1.213699
H	-2.353597	2.045874	-2.125376
H	-3.383673	3.179411	-1.230053
H	-1.627065	3.391363	-1.227228
C	-7.064084	-0.242337	0.054386
B	-7.734442	1.116230	-0.800896
B	-7.822070	0.991640	0.970149
B	-8.054167	-1.658770	-0.112138
B	-8.018073	-0.725454	1.396856
C	-7.943384	-0.485212	-1.347732
H	-6.993910	1.813613	-1.395065
B	-9.240943	1.504129	0.029436
B	-9.278912	0.557447	-1.475405
H	-7.128498	1.688885	1.626001
B	-9.428502	0.360249	1.390337
H	-7.542907	-2.704848	-0.281371
B	-9.473268	-1.155202	-1.052542
B	-9.563685	-1.288683	0.719954
H	-7.448109	-1.176806	2.329460
H	-9.626059	2.625518	0.064103
B	-10.329797	0.091971	-0.121345
H	-9.578271	0.947794	-2.551131
H	-9.952129	0.674329	2.408235
H	-9.905579	-1.923692	-1.840921
H	-10.179162	-2.158651	1.240779
H	-11.508342	0.207457	-0.198508
H	-7.353330	-0.766797	-2.210561
H	7.353330	0.766801	-2.210562

Table S8. Cartesian coordinates of the first excited state (S_1) fully optimized geometry in THF of *closo*-**PT** from B3LYP calculations (in Å)

Atom	x	y	z
C	7.993327	-1.362407	-0.549977
H	9.905569	-0.494389	2.628670
H	7.283708	-1.963802	1.774101
H	9.843899	-2.698396	0.427485
H	11.527520	-0.084845	0.051581
H	7.200375	0.995096	2.272761
H	9.821737	-1.390078	-2.227831
H	9.857078	2.224538	1.201762
H	7.235319	0.100263	-2.427597
H	7.219357	2.320124	-0.392497
H	9.864574	1.701898	-1.816494
B	7.924944	-1.190596	1.157855
B	9.405198	-0.328745	1.566761
B	9.434165	-1.594902	0.316594
B	7.876144	0.570253	1.400611
B	10.341545	-0.088541	0.060070
B	9.422802	-0.806513	-1.280103
B	9.374276	1.249634	0.728818
B	7.897937	0.093074	-1.452397
B	7.867258	1.352331	-0.199923
B	9.381997	0.951969	-1.035400
C	5.566987	-0.290730	0.022880
C	3.583269	-1.708374	-0.006952
C	4.760856	0.863009	0.013493
C	2.782314	-0.552459	-0.023683
H	3.139589	-2.697997	-0.007918
H	5.218187	1.844083	0.026201
C	7.068690	-0.145834	0.062536
C	2.404788	2.713546	1.240698
H	3.355047	3.254802	1.269406
H	1.597564	3.451811	1.241392
H	2.322672	2.111135	2.149370
C	2.446866	2.710392	-1.294986
H	1.641220	3.449744	-1.323713
H	3.398741	3.249799	-1.294556
H	2.392928	2.105617	-2.204223
C	4.961467	-1.567393	0.020548
H	5.570426	-2.462460	0.057045
C	3.383321	0.733001	-0.010146
C	1.046324	1.000162	-0.048053
C	2.325838	1.832697	-0.027512
C	1.352354	-0.400759	-0.046846
C	0.334640	-1.381352	-0.062470
H	0.584537	-2.437679	-0.060836
C	-0.261950	1.433756	-0.064044
H	-0.511814	2.489864	-0.065028
C	-0.971525	-0.949361	-0.076643
C	-1.279108	0.454863	-0.078655
C	-2.713359	0.607773	-0.099835
C	-3.514515	1.762994	-0.114341
C	-3.311283	-0.676933	-0.112633
C	-4.893034	1.620888	-0.136316
H	-3.068388	2.752566	-0.109217
C	-4.691856	-0.809027	-0.134735
C	-5.505924	0.341897	-0.137043
H	-5.521518	2.503412	-0.150538
H	-5.154753	-1.789326	-0.145403
C	-2.253662	-1.778429	-0.093032
C	-2.334892	-2.664561	-1.356837
H	-2.245808	-2.066278	-2.267694
H	-1.535968	-3.412457	-1.354134
H	-3.291563	-3.194531	-1.386319
C	-2.373698	-2.651060	1.177567
H	-2.310723	-2.042934	2.084131
H	-3.331762	-3.179376	1.184245
H	-1.575834	-3.399447	1.206696
C	-6.987938	0.218548	-0.156847
B	-7.802877	-1.095919	0.628085
B	-7.814548	-0.728857	-1.187499
B	-8.001337	1.341832	0.692545
B	-7.961573	1.072777	-1.138902
C	-8.377538	0.055513	1.738922
H	-7.147134	-1.998414	1.035601
B	-9.273917	-1.397135	-0.452227
B	-9.554587	-0.930622	1.232177
H	-7.176430	-1.361397	-1.965629
B	-9.387703	0.058422	-1.496706
H	-7.495778	2.314889	1.148733
B	-9.700388	0.863690	1.279945
B	-9.506621	1.457031	-0.377290
H	-7.437638	1.840988	-1.879278
H	-9.645292	-2.468302	-0.815243
B	-10.412617	-0.061899	-0.074279
H	-10.021124	-1.698461	2.010250
H	-9.851933	0.049651	-2.593733
H	-10.281244	1.504494	2.095146
H	-10.047651	2.471470	-0.685859
H	-11.595495	-0.155742	-0.179104
H	-7.982065	0.061357	2.746189
H	7.454826	-2.199868	-0.971952
